# Metabolic Response of Triple-Negative Breast Cancer to Folate Restriction

**DOI:** 10.3390/nu13051637

**Published:** 2021-05-13

**Authors:** Michael F. Coleman, Ciara H. O’Flanagan, Alexander J. Pfeil, Xuewen Chen, Jane B. Pearce, Susan Sumner, Sergey A. Krupenko, Stephen D. Hursting

**Affiliations:** 1 Department of Nutrition, University of North Carolina, Chapel Hill, NC 27599, USA; mcoleman@unc.edu (M.F.C.); oflanach@gmail.com (C.H.O.); pfeilal@live.unc.edu (A.J.P.); xuewen@live.unc.edu (X.C.); japearce@wakehealth.edu (J.B.P.); susan_sumner@unc.edu (S.S.); sergey_krupenko@unc.edu (S.A.K.); 2 Nutrition Research Institute, University of North Carolina, Kannapolis, NC 28081, USA; 3 Lineberger Comprehensive Cancer Center, University of North Carolina, Chapel Hill, NC 27599, USA

**Keywords:** one-carbon metabolism, triple-negative breast cancer, mitochondria, glycolysis, dietary folate, metabolomics

## Abstract

Background: Triple-negative breast cancers (TNBCs), accounting for approximately 15% of breast cancers, lack targeted therapy. A hallmark of cancer is metabolic reprogramming, with one-carbon metabolism essential to many processes altered in tumor cells, including nucleotide biosynthesis and antioxidant defenses. We reported that folate deficiency via folic acid (FA) withdrawal in several TNBC cell lines results in heterogenous effects on cell growth, metabolic reprogramming, and mitochondrial impairment. To elucidate underlying drivers of TNBC sensitivity to folate stress, we characterized in vivo and in vitro responses to FA restriction in two TNBC models differing in metastatic potential and innate mitochondrial dysfunction. Methods: Metastatic MDA-MB-231 cells (high mitochondrial dysfunction) and nonmetastatic M-Wnt cells (low mitochondrial dysfunction) were orthotopically injected into mice fed diets with either 2 ppm FA (control), 0 ppm FA, or 12 ppm FA (supplementation; in MDA-MB-231 only). Tumor growth, metabolomics, and metabolic gene expression were assessed. MDA-MB-231 and M-Wnt cells were also grown in media with 0 or 2.2 µM FA; metabolic alterations were assessed by extracellular flux analysis, flow cytometry, and qPCR. Results: Relative to control, dietary FA restriction decreased MDA-MB-231 tumor weight and volume, while FA supplementation minimally increased MDA-MB-231 tumor weight. Metabolic studies in vivo and in vitro using MDA-MB-231 cells showed FA restriction remodeled one-carbon metabolism, nucleotide biosynthesis, and glucose metabolism. In contrast to findings in the MDA-MB-231 model, FA restriction in the M-Wnt model, relative to control, led to accelerated tumor growth, minimal metabolic changes, and modest mitochondrial dysfunction. Increased mitochondrial dysfunction in M-Wnt cells, induced via chloramphenicol, significantly enhanced responsiveness to the cytotoxic effects of FA restriction. Conclusions: Given the lack of targeted treatment options for TNBC, uncovering metabolic vulnerabilities that can be exploited as therapeutic targets is an important goal. Our findings suggest that a major driver of TNBC sensitivity to folate restriction is a high innate level of mitochondrial dysfunction, which can increase dependence on one-carbon metabolism. Thus, folate deprivation or antifolate therapy for TNBCs with metabolic inflexibility due to their elevated levels of mitochondrial dysfunction may represent a novel precision-medicine strategy.

## 1. Background

Folate (vitamin B9) is an essential nutrient that is integral to cellular function, as reduced folates are requisite coenzymes in the one-carbon transfer linked to amino acid and nucleotide metabolism [1]. Dietary folate deficiency causes several developmental disorders, most notably neural tube defects, many of which are prevented by adequate folate supplementation [1,2]. Likewise, epidemiological studies suggest that sufficient dietary folate diminishes cancer initiation, though this effect appears to be cancer type specific [3,4,5]. Genetic polymorphisms in several folate enzymes, most notably methylenetetrahydrofolate reductase (MTHFR), have also been associated with increased risk of several cancers [6,7,8], underscoring the role of folate metabolism in tumorigenesis.

Conversely, excess folate is likely to contribute to the growth of initiated cancers [9,10,11], while folate analogs (antifolates) inhibit proliferation of cancer cells [12]. The antifolate methotrexate, a dihydrofolate reductase inhibitor, has been used as a chemotherapeutic agent for more than 60 years [13]. Recent studies indicate that methotrexate treatment, in combination with cyclophosphamide and/or fluorouracil, may specifically benefit patients with triple-negative breast cancers (TNBCs) in advanced disease either as an adjuvant [14,15] or as part of metronomic treatment protocols [16]. TNBCs, which account for ~15% of breast cancers [14,15], currently lack FDA-approved targeted therapies, leaving systemic chemotherapy as the standard-of-care treatment for both early and advanced disease [17]. TNBCs tend to exhibit higher recurrence and metastasis rates compared with other breast cancer subtypes [18,19].

Folate has a pleiotropic effect at the cellular and whole organism levels due to participation in a host of key biological processes [1]. Among them, folate-dependent mitochondrial homeostasis is an area of growing interest [20,21]. Specifically, genetic disruption of folate metabolism results in significant mitochondrial dysfunction [20,21,22], with enhanced mitochondrial one-carbon metabolism playing an important role in the response to cellular energy crises, such as hypoxia [23] or limited glucose supply [24]. Indeed, folate-dependent serine metabolism is critical to maintenance of redox homeostasis when electron transport chain (ETC) activity is inhibited pharmacologically or by hypoxia [25]. Thus, one-carbon metabolism is at the nexus of several metabolic branches relevant to biosynthetic processes, redox defense, and bioenergetics, all of which are essential for mitochondrial health [26]. Accordingly, folate deficiency and/or dysregulation of folate metabolism produces conditions under which cellular metabolic plasticity and adaptation are required for survival. Towards this end, we have shown that in vitro folic acid (FA) deficiency in several TNBC cell lines produces heterogenous effects on cell growth and migration, metabolic reprogramming, mitochondrial impairment, reduced energy status, and altered pentose phosphate pathway (PPP) metabolism [27,28].

Tumors with pre-existing mitochondrial defects or impaired mitochondrial function may be especially sensitive to manipulation of folate metabolism, such as dietary folate depletion or antifolate therapies [20,21,25]. Overall, a better understanding of the response of TNBCs to folate deprivation and antifolates has potential to aid in the identification of patient populations who may benefit most from the inclusion of neoadjuvant or adjuvant antifolates. Dietary FA may be required for metastasis-related processes, including epithelial-to-mesenchymal transition (EMT) and efficient lung colonization in A549 lung cancer cells [29]. Moreover, MMTV-PyMT transgenic mice supplemented with excess FA exhibited enhanced tumor growth [30]. These findings imply an important role for folate in cancer progression and metastasis, although mechanisms underlying such a role are not fully understood. To identify underlying drivers of TNBC responsiveness to folate deprivation, we characterized in vivo and in vitro metabolic responses to FA manipulation in two TNBC models differing in metastatic potential and innate mitochondrial dysfunction.

## 2. Methods

### 2.1. Animal Studies

All animal studies were approved by the Institutional Animal Care and Use Committee at the University of North Carolina at Chapel Hill and were performed by the Animal Studies Core Facility at the University of North Carolina at Chapel Hill. Female 8-week-old C57Bl/6N mice (12–14 mice per treatment group) were purchased from Charles River Laboratories. Female 8-week-old C57Bl/6 B6.Cg-Foxn1^nu^/J nude mice (10–14 mice per treatment group) were purchased from the Jackson Laboratory.

Mice were allowed to acclimate to the control diet (which includes 2 ppm FA; Research Diets # D12450J) for 12–16 weeks before orthotopic transplantation of tumor cells. Mice were then randomized to either continue on the same control regimen, or switch to modified D12450J diet with FA levels of 0 ppm (No FA) or (for the MDA-MB-231 model only) 12 ppm (Supp FA). These levels were chosen to emulate the broad range of FA observed in human diets spanning from FA-deficient intakes to abundant FA intakes achieved through fortification of processed grain products and/or FA supplement use. Given the similarity in tumor growth between control and Supp FA diet groups seen in the MDA-MB-231 model, the Supp FA group was omitted from the M-Wnt model. Three weeks after diet switch, C57Bl/6 nude mice were injected with 1 × 10^6^ luciferase-labelled MDA-MB-231 human TNBC cells (purchased from ATCC, Gaithersburg, MD; metastatic, high innate mitochondrial dysfunction) in a 1:1 suspension of PBS:Geltrex (Thermo Fischer, Waltham, MA, USA) in the 4th mammary fat pad. In addition, C57Bl/6N mice were injected with 5 × 10^4^ murine M-Wnt cells [31] nonmetastatic, low innate mitochondrial dysfunction) in the 4th mammary fat pad. MDA-MB-231 growth was monitored by bioluminescent imaging using an IVIS Spectrum (Waltham, MA, USA) and by palpation with electronic calipers. M-Wnt tumor growth was monitored by palpation with electronic calipers. In both models, body composition was assessed one week prior to study termination (4 weeks post-injection for MDA-MB-231 model and 3 weeks for M-Wnt model) by magnetic resonance (EchoMRI, Houston, TX, USA). Mice were euthanized using CO_2_ followed by cervical dislocation, and tumor, liver, and serum were collected. Tumor mass and volume were determined following excision. Tumor and liver were divided and flash frozen or fixed in formalin and paraffin embedded.

### 2.2. Metabolomics Analysis

Metabolomics analysis was performed by Metabolon (Morrisville, NC). Metabolites were isolated from a randomized selection of 6 frozen samples of liver and tumor per diet group collected from the MDA-MB-231 and M-Wnt tumor transplant studies, using methanol with vigorous shaking for 2 min followed by centrifugation. The resulting extract was divided and analyzed using (i) reverse-phase (RP)/UPLC–MS/MS with positive-ion-mode electrospray ionization (ESI); and (ii) RP/UPLC–MS/MS with negative-ion-mode ESI, (iii) HILIC/UPLC–MS/MS with negative-ion-mode ESI. Compounds were identified by comparison to library entries of purified standards or recurrent unknown entities, based on retention time/index (RI), mass to charge ratio (*m/z)*, and chromatographic data (including MS/MS spectral data). Peaks were quantified using area under the curve, and normalized to the average of the control diet group. Principle component analysis (PCA), orthogonal partial least squares discriminant analysis (OPLS-DA), and random forest (RF) classification were conducted using soft independent modeling by class analogy (SIMCA) software. All metabolite levels detected are shown in supplementary files, including key folate cycle metabolites 5mTHF and DHF.

### 2.3. Cell Culture Studies

Unless otherwise noted, all cells were maintained in RPMI-1640 with 10% FBS, 11 mM glucose, 2.2 μM FA, 2 mM L-glutamine and 100 U/mL penicillin/streptomycin. For FA withdrawal, M-Wnt and MDA-MB-231 cells were incubated with FA-free RPMI-1640 supplemented with 10% dialyzed FBS. Culture in these conditions for 6 weeks resulted in growth arrest of MDA-MB-231 cells hence, 3 weeks was used for long term depletion of FA, reflecting a similar number of cell doublings for both lines. Cells were incubated with 2-deoxyglucose (2DG; 50 mM), polydatin (polyD; 10 µM), or 3-bromopyruvate (3BrPyr; 20 µM) for 24 h, and cytotoxicity was determined by MTT (3-(4,5-dimethylthiazol-2-yl)-2,5-diphenyl tetrazolium bromide) assay.

### 2.4. Flow Cytometry Analysis

All flow cytometry was performed using a CytoFlex cytometer (Beckman Coulter, Brea, CA, USA). Cells were incubated for 30 min at 37 °C in PBS with MitoSox Red (for mitochondrial superoxide), MitoTracker Green (for mitochondrial mass), or the fluorescent glucose analog 2NBDG (for glucose uptake) as per the manufacturer’s guidelines and harvested using PHEM buffer (all Thermo Fisher, Waltham, MA, USA).

### 2.5. Extracellular Flux Analysis

The cellular oxygen consumption rate (OCR), a measure of mitochondrial function, was determined using a XF96 Seahorse Metabolic Flux Analyzer (Agilent Seahorse Technologies, Santa Clara, CA, USA). Cells were seeded into XF96 Seahorse cell culture plates at a density of 1 × 10^4^ cells/well for M-Wnt and 1.5 × 10^4^ cells/well for MDA-MB-231 18 h prior to assay. Cells were incubated in assay media (serum-free RPMI-1640 media with 10 mM glucose, 2 mM glutamine and 1 mM pyruvate, without bicarbonate, pH 7.4) in a non-CO_2_ incubator for one hour prior to analysis. Oligomycin (1.0 µM), carbonyl cyanide-4-(trifluoromethoxy)phenylhydrazone (FCCP; 1.0 µM), and rotenone/antimycin A (0.5 µM) were added sequentially, and OCR was measured. Measurements were normalized by total protein amount using a bicinchoninic acid protein assay (Thermo Fisher, Waltham, MA, USA) and expressed as relative OCR.

### 2.6. RT-qPCR Analysis

RNA was isolated from tissue using E.Z.N.A HP total RNA isolation kit (Omega Biotech, Norcross, GA, USA) and cDNA reverse transcribed using ABI High-Capacity cDNA Reverse Transcription Kit (Thermo Fisher, Waltham, MA, USA). Human- and mouse-specific gene primer sequences were obtained from primerbank [32] and are listed in Table S1. qPCR was performed using SsoAdvanced Universal SYBR Green Supermix (Bio-Rad, Hercules, CA, USA), and relative expression calculated by 2^−ΔΔCT^ as previously described [33].

### 2.7. Statistical Analysis

Comparisons of two groups were conducted using Student’s *t*-test, and comparisons of three or more were conducted using one-way ANOVA. For metabolomics analysis relative abundance of metabolites was determined by ANOVA. Random forest analysis was conducted on the 30 metabolites with highest variable importance in projection (VIP) score, and mean decrease accuracy visualized. PCA and OPLS-DA were visualized for each analysis. Multiple hypothesis correction was performed using Benjamini–Hochberg correction. Groups were considered different if P (or adjusted P where multiple hypotheses were tested) was less than 0.05.

## 3. Results

### 3.1. FA Restriction Inhibits Growth of Transplanted MDA-MB-231 Tumors

**Figure 1:**
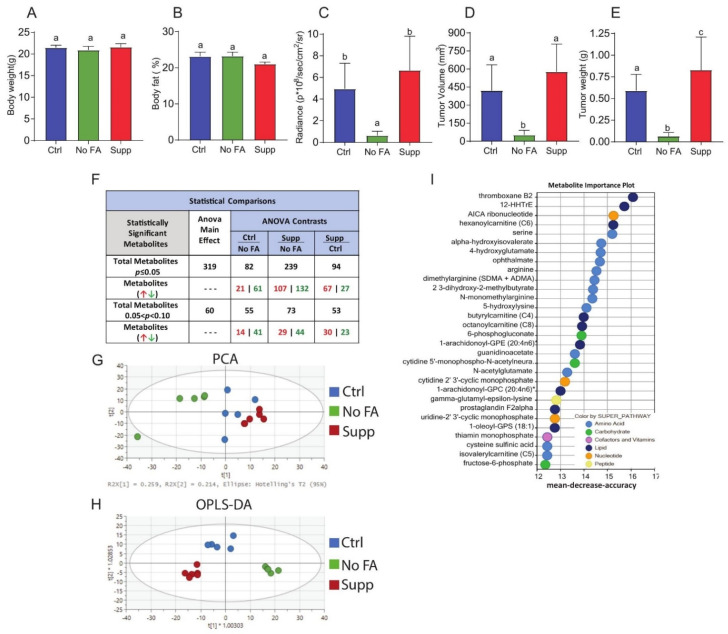
Dietary folate restriction inhibits growth of MDA-MB-231 tumors and alters metabolomic profiles. Body weight (**A**) and body fat% (**B**) of mice fed control (Ctrl, 2 ppm FA), FA-deficient (No FA, 0 ppm FA) or FA-supplemented (Supp, 12 ppm FA) diets (n = 12–14/group). IVIS bioluminescence imaging of luciferase-labelled MDA-MB-231 tumors (**C**) (n = 13–14/group). Ex vivo volume (**D**) and weight (**E**) of MDA-MB-231 tumors at the endpoint of the experiment (n = 12–13/group). Summary of metabolome analysis (**F**) (n = 6 mice per diet). PCA and OPLS-DA of tumors from three diet groups (**G**,**H**) (n = 6/group). RF classification importance plot of the top 30 ranking metabolites and associated key metabolic pathways (**I**) (n = 6/group). All bar charts represent the mean ± SD, and statistical significance (*p <* 0.05) was determined by one-way ANOVA (**A**–**F**), different letters indicate significant differences.

To determine the contribution of dietary FA to breast cancer progression, MDA-MB-231 cells were injected into the 4th mammary fat pad of C57Bl/6 nude mice fed a diet containing 0, 2, or 12 ppm FA. The three diet groups showed no difference in body weight or percent body fat (Figure 1A,B). FA-restricted (0 ppm FA) mice, relative to mice fed diets containing 2 or 12 ppm FA, showed significantly smaller MDA-MB-231 tumors as determined by in vivo imaging and ex vivo tumor volume and mass measurements (Figure 1C–E). The mice receiving the 12 ppm FA regimen, relative to the 2 ppm FA group, showed a significant increase in mean tumor weight (Figure 1E) but not tumor size via in vivo imaging (Figure 1C) or ex vivo tumor volume (Figure 1D).

Gene expression analysis of tumors revealed no diet-dependent reduction in transcripts associated with total leukocytes (CD45, *Ptprc*), macrophages (F4/80, *Emr1*), total T cells (Cd3e), or cytotoxic T cells (Cd8b), indicating that FA restriction effects on tumor growth were not driven by immunodeficiency in FA-deprived C57Bl/6 nude mice (data not shown).

### 3.2. FA Restriction Alters Metabolomic Profiles of MDA-MB-231 Tumors

Untargeted metabolomic analysis of MDA-MB-231 tumors (Figure 1F–H, Table S2) revealed that dietary FA restriction induced profound metabolic alterations. Out of 760 total named metabolites, significant between-group differences were observed for 82 metabolites in the 2 ppm versus 0 ppm dietary FA groups, 94 in the 12 ppm versus 2 ppm FA diet groups, and 239 in the 12 ppm versus 0 ppm FA diet groups (Figure 1F). Both unsupervised PCA (Figure 1G) and supervised OPLS-DA (Figure 1H) demonstrated clustering of metabolic profiles based on dietary FA concentration. RF classification and VIP scores of named metabolites in tumor tissues of mice fed diets containing 0, 2, or 12 ppm FA revealed several responsive metabolic pathways, including metabolism of amino acids, nucleotides, carbohydrates, and lipids (Figure 1I, and Table S3).

### 3.3. FA Restriction Alters One-Carbon Metabolism in MDA-MB-231 Tumors

**Figure 2:**
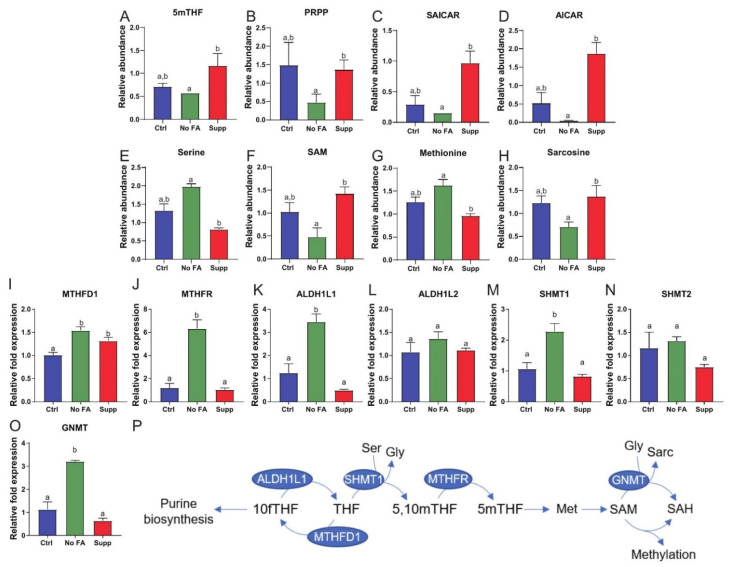
Dietary FA modulates tumor one-carbon metabolism. Comparison of one-carbon pathway metabolites in MDA-MB-231 tumors from control (Ctrl, 2 ppm FA), FA-deficient (No FA, 0 ppm FA), or FA-supplemented (Supp, 12 ppm FA) diets (n = 6 samples/group) (**A**–**H**). Relative abundance of 5mTHF (**A**), PRPP (**B**), SAICAR (**C**), AICAR (**D**), serine (**E**), SAM (**F**), methionine (**G**), sarcosine (**H**). Expression of MTHFD1 (**I**), MTHFR (**J**), ALDH1L1 (**K**), ALDH1L2 (**L**), SHMT1 (**M**), SHMT2 (**N**), and GNMT (**O**) measured by qPCR in tumors from each diet group (n = 5/group). Pathway of cytosolic folate enzymes evaluated in this study (**P**). One-way ANOVA used to determine statistical significance (*p* < 0.05), different letters indicate significant differences.

The examination of several folate-related pathways at a metabolite level indicated that FA restriction impacts de novo purine biosynthesis in MDA-MB-231 tumors. Tumoral levels of 5-methyltetrahydrofolate (5MeTHF), phosphoribosyl pyrophosphate (PRPP, the starting point of purine biosynthesis), and the intermediates phosphoribosylaminoimidazolesuccinocarboxamide (SAICAR) and 5-aminoimidazole-4-carboxamide ribonucleotide (AICAR), were all decreased in the FA-restricted group relative to the FA-supplemented group (Figure 2A–D). Serine, methionine, and sarcosine were each higher in the FA-restricted group than the FA-supplemented group, while S-adenosylmethionine (SAM) was lower (Figure 2E–H). FA restriction, relative to control, led to significantly increased mRNA expression of several folate metabolism enzymes in MDA-MB-231 tumors, including cytosolic MTHFD1, MTHFR, SHMT1, ALDH1L1, and GNMT, but not mitochondrial SHMT2 and ALDH1L2 (Figure 2I–P). FA supplementation, relative to control, had no significant effect on expression of these enzymes, with the exception of MTHFD1, which was significantly increased (Figure 2I–P).

### 3.4. FA Restriction Enhances Glycolysis and PPP Metabolism in MDA-MB-231 Cells In Vivo and In Vitro

**Figure 3:**
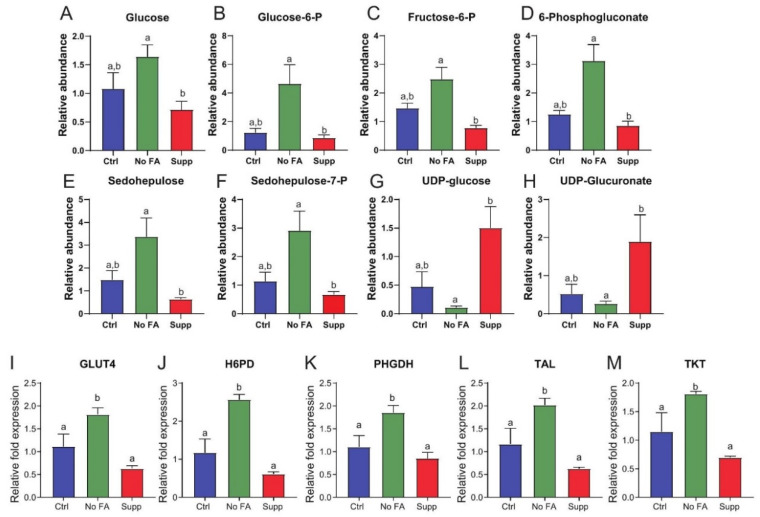
Dietary FA modulates glucose metabolism in MDA-MB-231 tumors. Comparison of glucose-linked metabolites in MDA-MB-231 tumors from control (Ctrl, 2 ppm FA), FA-deficient (No FA, 0 ppm FA), or FA-supplemented (Supp, 12 ppm FA) diets (n = 6 samples/group) (**A**–**H**). Relative abundance of glucose (**A**), glucose-6-P (**B**), fructose-6-P (**C**), 6-phosphogluconate (**D**), sedohepulose (**E**), sedohepulose-7-P (**F**), UDP-glucose (**G**), UDP-glucuronate (**H**). Expression of GLUT4 (**I**), H6PD (**J**), PHGDH (**K**), TAL (**L**), and TKT (**M**) measured by qPCR in tumors from each diet group (n = 5/group). One-way ANOVA used to determine statistical significance (*p <* 0.05), different letters indicate significant differences.

Glucose-6 phosphate (G6P)-dependent glycolysis and the PPP contribute ATP and NADPH required for cellular redox homeostasis and anabolic processes, as well as anabolic carbon in the form of pyruvate, PRPP, and serine (Figure 3A). Metabolomic analysis of MDA-MB-231 tumors showed that dietary FA restriction enhanced glycolysis and PPP metabolism (Figure 3A–H and Table S2). Specifically, MDA-MB-231 tumors from FA-restricted mice, relative to FA-supplemented mice, revealed significant increases in glucose, glucose-6 phosphate, and fructose-6 phosphate, along with upregulation of PPP intermediates, including 6-phosphogluconate, sedoheptulose, and sedoheptulose-7-phosphate (Figure 3A–F). In addition, numerous metabolites of glucose-dependent pathways of sugar nucleotide derivatives, including UDP-glucose and UDP-galactose, were decreased in tumors from FA-restricted, relative to FA-supplemented, mice (Figure 3G,H). Tumoral expression of several PPP- and glycolysis-associated transcripts, including GLUT4, H6PD, TKT, TAL, and PHGDH, were significantly upregulated following FA restriction relative to control (Figure 3I–M). No significant differences in these transcripts were observed between tumors from mice fed diets containing 2 ppm versus 12 ppm FA (Figure 3I–M).

**Figure 4:**
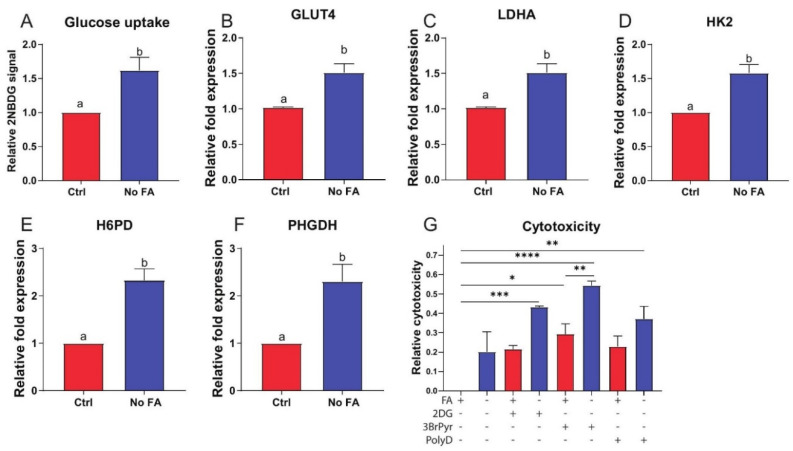
Effect of FA on glucose metabolism in cultured MDA-MB-231 cells. MDA-MB-231 cells cultured in standard (2.2 µM) or FA-deficient (0 µM) medium for 3 weeks prior to analysis. Glucose uptake determined by 2NBDG staining (n = 3/group) (**A**). Expression of GLUT4 (**B**), LDHA (**C**), HK2 (**D**), H6PD (**E**), and PHGDH (**F**) determined by qPCR (n = 3/group). Cytotoxicity following withdrawal of FA in the presence of 50 mM 2-deoxyglucose (2DG), 30 µM 3-bromopryuvate (3BrPyr), or 10 µM polydatin (PolyD) for 48 h as determined by MTT assay (n = 4/group) (**G**). Student’s *t*-test (**A**–**F**) or one-way ANOVA (**G**) used to determine statistical significance (*p <* 0.05) between groups. Different letters indicate significant differences determined by Student’s *t*-test. Asterix’s indicate significant differences determined by one-way ANOVA (* *p* < 0.05, ** *p* < 0.01, *** *p* < 0.001, **** *p* < 0.0001).

To confirm that this metabolic shift was accompanied by an increased requirement for glucose, we assayed in vitro uptake of the fluorescent glucose analog 2NBDG in cultured MDA-MB-231 cells using flow cytometry. Consistent with the gene expression data, glucose uptake was increased following FA withdrawal for 3 weeks (Figure 4A). mRNA levels of several glycolytic enzymes were elevated in MDA-MB-231 cells cultured without (versus with) FA for 3 weeks (Figure 4B–F).

To test whether MDA-MB-231 cells redirect glycolytic carbon towards one-carbon metabolism via the PPP and serine production in response to FA restriction, we assessed the effect of glycolytic inhibitors 2-deoxyglucose (2DG) and 3-bromopyruvate (3BrPyr), and the PPP inhibitor polydatin (PolyD), on MDA-MB-231 cells cultured in the presence or absence of FA. PolyD and 2DG both induced significant cytotoxicity only when combined with FA withdrawal (Figure 4G). 3BrPyr induced significant cytotoxicity alone, which was enhanced by withdrawal of FA (Figure 4G).

### 3.5. FA Restriction Enhances Mitochondrial Dysfunction in MDA-MB-231 Cells In Vivo and In Vitro

**Figure 5:**
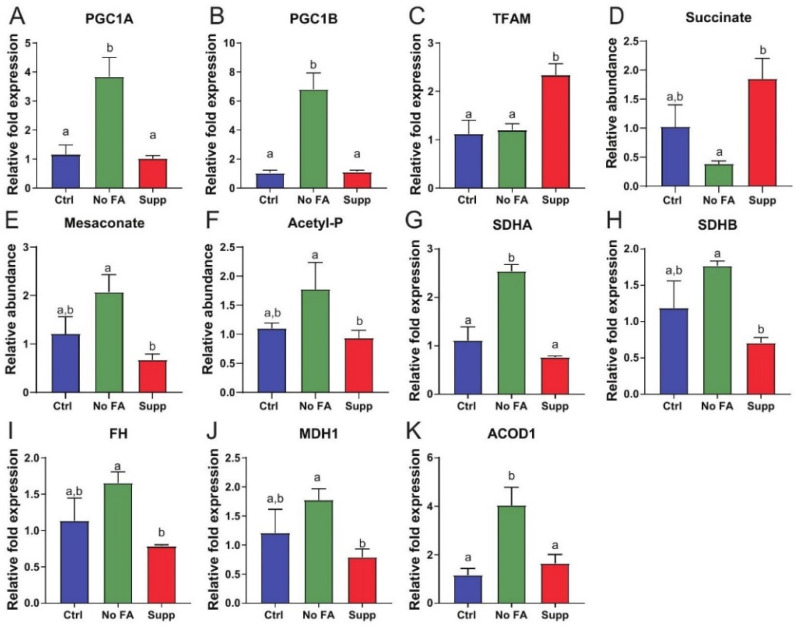
FA restriction enhances mitochondrial dysfunction in MDA-MB-231 tumors. Expression of PGC1α (**A**), PGC1ß (**B**), and TFAM (**C**) measured by qPCR in tumors from each diet group (n = 5/group). Relative abundance of succinate (**D**), mesaconate (**E**), acetyl-P (**F**) MDA-MB-231 tumors from control (Ctrl, 2 ppm FA), FA-deficient (No FA, 0 ppm FA), or FA-supplemented (Supp, 12 ppm FA) diets (n = 6 samples/group). Expression of SDHA (**G**), SDHB (**H**), FH (**I**) and MDH1 (**J**), and ACOD1 (**K**) measured by qPCR in tumors from each diet group (n = 5/group). One-way ANOVA used to determine statistical significance (*p <* 0.05), different letters indicate significant differences.

Metabolomic analysis indicated that transplanted MDA-MB-231 tumors experience increased oxidative stress and/or display mitochondrial dysfunction (Table S2). We thus assayed markers of mitochondrial biogenesis by qPCR and found PGC1α, PGC1ß were upregulated in tumors from mice fed diet with 0 ppm versus 12 ppm FA (Figure 5A–C), while TFAM was lower (Figure 5A–C).

Succinate, a key TCA intermediate, was reduced in tumors from FA-restricted mice relative to FA-supplemented mice (Figure 5D and Table S2). Additionally, we observed accumulation of carbon in metabolites prior to entry into the TCA cycle, indicated by increases in several metabolites including mesaconate and acetylphosphate (Figure 5E,F and Table S2). Furthermore, upregulation of SDHA, SDHB, fumarase (FH), and malate dehydrogenase (MDH) was observed in MDA-MB-231 tumors following FA restriction compared with tumors from mice fed diet with 12 ppm FA (Figure 5G–J).

**Figure 6:**
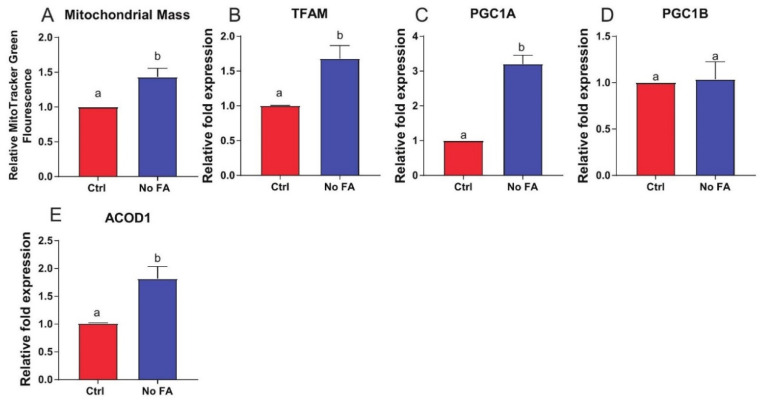
Mitochondrial response in cultured MDA-MB-231 cells to medium FA withdrawal. MDA-MB-231 cells were cultured with 0 or 2.2 µM FA for 3 weeks prior to assessment of mitochondrial characteristics. Mitochondrial mass determined by MitoTracker Green (n = 3/group) (**A**). Expression of TFAM (**B**), PGC1α (**C**), PGC1ß (**D**), and ACOD1 (**E**) determined by qPCR (n = 3/group). Student’s *t*-test was used to determine statistical significance between groups (*p <* 0.05), different letters indicate significant differences.

In MDA-MB-231 cells following 3 weeks of culturing in media deficient versus replete in FA, markers of mitochondrial biogenesis/dysfunction, including mitochondrial mass and mRNA expression of TFAM, PGC1α (but not PGC1ß), and ACOD1 were increased (Figure 6A–E).

### 3.6. FA Restriction Enhances Growth of Transplanted M-Wnt Mammary Tumors

**Figure 7:**
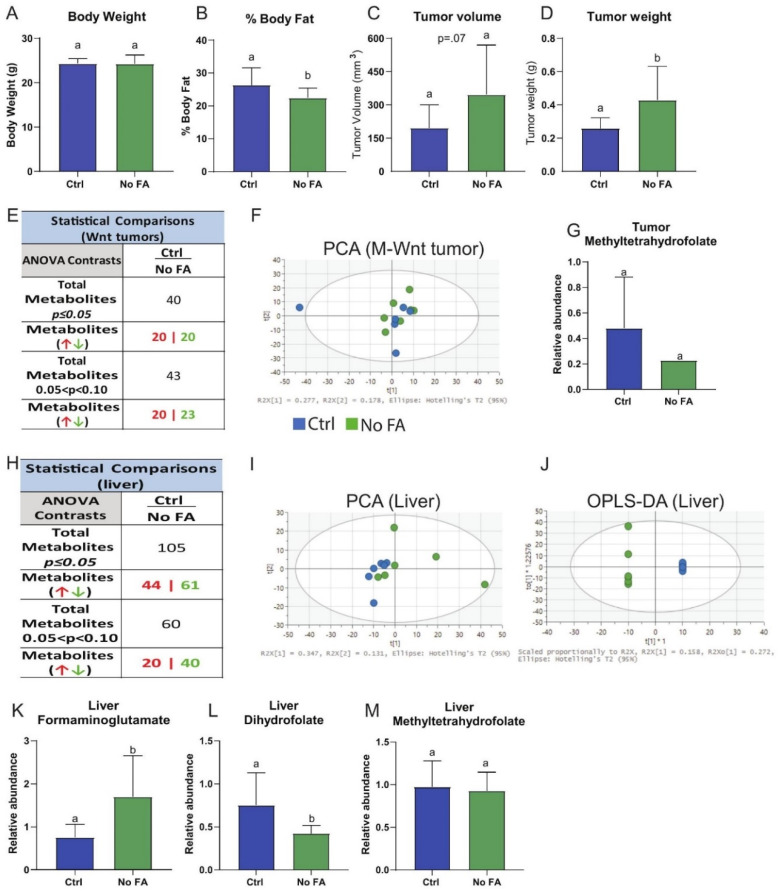
Dietary FA restriction enhances growth of orthotopically transplanted M-Wnt mammary tumors and minimally effects tumor metabolomic profile. Body weight of mice fed different folate diets for 3 weeks (**A**) (n = 10–14/group). Body composition determined by echo MRI (**B**) (n = 10–14/group). Ex vivo tumor volume (**C**) and weight (**D**) (n = 9–13/group). Summary of tumor metabolomics (**E**), PCA of tumor metabolomics (**F**) (n = 6/group). Tumor metabolite levels of 5-mTHF (**G**) (n = 6–7/group), different letters indicate significant differences.

Our previous in vitro studies showed that metabolic reprograming of folate-deprived M-Wnt cells induced a less aggressive cancer phenotype [27]. To determine whether FA deprivation and resulting mitochondrial stress impacts the biology of a nonmetastatic TNBC model with low innate (prior to any FA treatment) mitochondrial dysfunction [31,34], we examined orthotopically transplanted M-Wnt tumor growth in mice fed control (2 ppm FA) or FA-restricted (0 ppm FA) diet. FA restriction did not significantly alter mean body weight but did decrease percent body fat (Figure 7A,B). Tumors from FA-restricted mice relative to control mice were approximately 2-fold higher in volume (*p* = 0.07; Figure 7C) and weight (*p <* 0.05; Figure 7D).

### 3.7. FA Restriction Minimally Alters M-Wnt Tumor Metabolomic Profiles, Glycolysis, and PPP Metabolism

Untargeted metabolomics analysis on M-Wnt tumors from mice fed 0 ppm versus 2 ppm FA diets showed that of the 760 named metabolites, 40 displayed diet-dependent differences (Figure 7E,F, Table S4). This was approximately half of the metabolite differences observed in MDA-MB-231 (FA-restricted relative to control; Figure 1F). FA restriction, relative to control, did not alter M-Wnt tumor 5-mTHF levels (Figure 7G), glycolysis, PPP, or one-carbon metabolism, in terms of metabolite levels (Table S4).

Given the minimal effect of FA restriction on metabolic reprogramming observed in M-Wnt tumors, untargeted metabolomic analysis was also conducted on livers from the C57BL/6N mice fed control or FA-deficient diets and injected with M-Wnt tumor cells (Table S5). Out of 760 total named metabolites, significant between-group differences were observed for 105 liver metabolites (Figure 7H, Table S5), and clustering was displayed (Figure 7I,J). In liver samples, formiminoglutamate (FIGLU), the intermediate of folate-dependent histidine degradation commonly used as a marker of folate deficiency [35], was increased in the 0 ppm FA diet group relative to control (Figure 7K) while dihydrofolate (DHF; Figure 7L), but not 5-mTHF (Figure 7M), was decreased. These findings indicate that FA restriction induced expected systemic metabolic changes.

**Figure 8:**
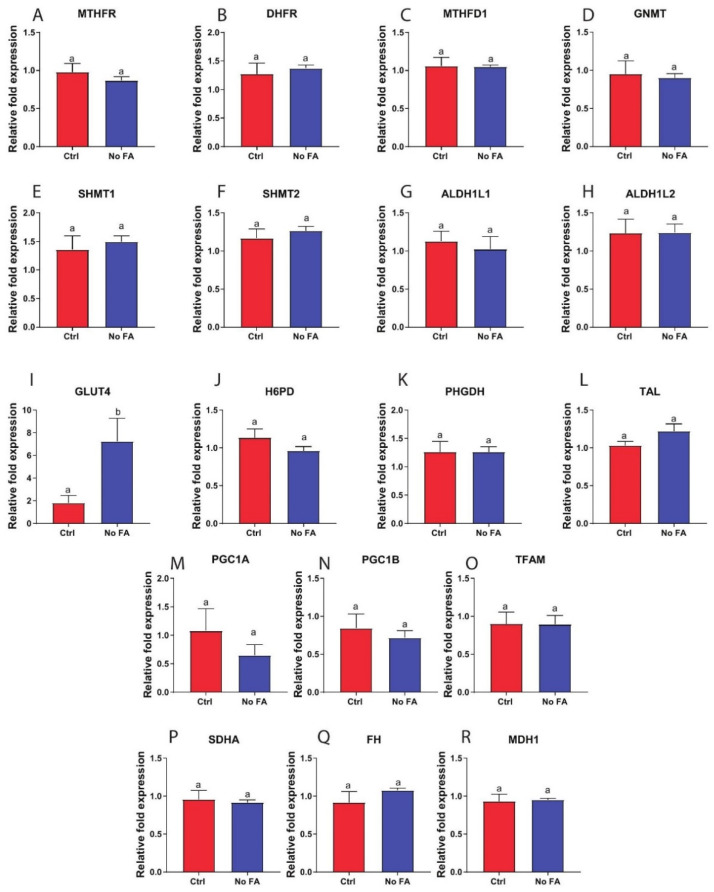
M-Wnt tumor response to FA withdrawal. M-Wnt tumor expression of MTHFR (**A**), DHFR (**B**), MTHFD1 (**C**), GNMT (**D**), SHMT1 (**E**), SHMT2 (**F**), ALDH1L1 (**G**), ALDH1L2 (**H**), GLUT4 (**I**), H6PD (**J**), PHGDH (**K**), TAL (**L**), PGC1A (**M**), PGC1B (**N**), TFAM (**O**), SDHA (**P**), FH (**Q**), and MDH1 (**R**) determined by qPCR (n = 5/group). Student’s *t*-test was used to determine statistical significance between groups (*p <* 0.05), different letters indicate significant differences.

Expression of folate enzymes in M-Wnt tumors (including MTHFR, DHFR, MTHFD1, GNMT, SHMT1, SHMT2, ALDH1L1, and ALDH1L2; Figure 8A–H), and glucose-metabolizing enzyme expression (including H6PD, PHGDH, TAL, PGC1a, PGC1b, TFAM, SDHA, FH, and MDH1; Figure 8J–R) were not altered by FA restriction. By exception, GLUT4 was significantly increased in response to FA restriction (Figure 8I). These findings contrasted with the metabolic reprogramming observed with FA restriction on MDA-MB-231 tumors (Figure 1, Figure 2, Figure 3, Figure 4, Figure 5 and Figure 6) and indicate that M-Wnt tumors may be more tolerant than MDA-MB-231 tumors to metabolic stress in response to FA restriction.

### 3.8. Innate Mitochondrial Dysfunction Predicts Sensitivity to FA Restriction

**Figure 9:**
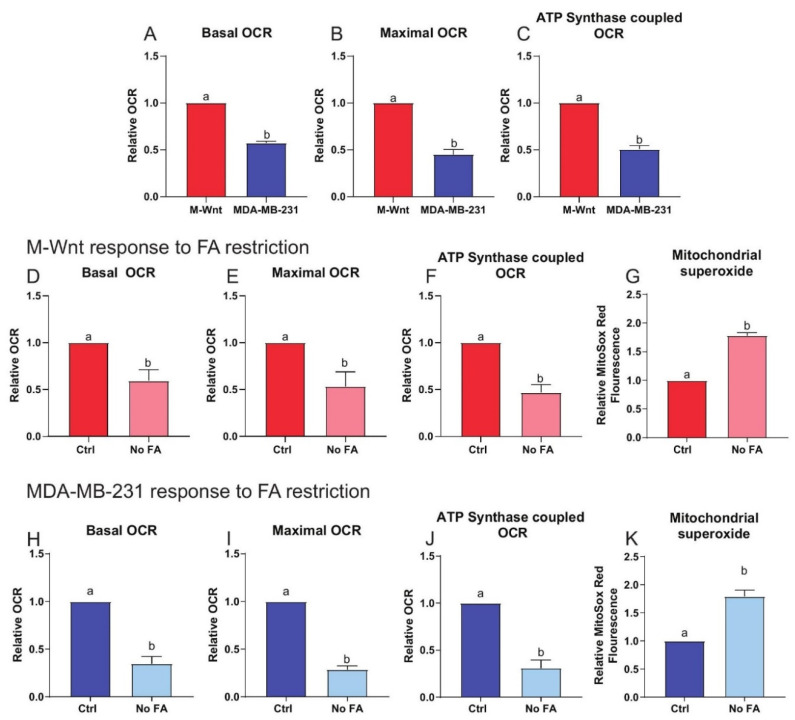
Innate mitochondrial dysfunction underpins response to FA restriction. Basal (**A**), maximal (**B**), and ATP synthase-coupled (**C**) OCR determined in M-Wnt cells and MDA-MB-231 cells (n = 3/group). Basal (**D**), maximal (**E**), and ATP synthase-coupled (**F**) OCR determined in M-Wnt cells following 3 weeks of culture with or without FA (n = 3/group). Mitochondrial superoxide production (**G**) determined using MitoSox Red in M-Wnt cells following 3 weeks of culture with or without FA (n = 3/group). Basal (**H**), maximal (**I**), and ATP synthase-coupled (**J**) OCR determined in MDA-MB-231 cells following 3 weeks of culture with or without FA (n = 3/group). Mitochondrial superoxide production (**K**) determined using MitoSox Red in MDA-MB-231 cells following 3 weeks of culture with or without FA (n = 3/group). Student’s *t*-test (**A**–**K**) used to determine significance (*p <* 0.05), different letters indicate significant differences.

We compared the innate mitochondrial activity of MDA-MB-231 and M-Wnt cells. MDA-MB-231 cells displayed approximately 50% lower basal, maximal, and ATP synthase-coupled OCR than M-Wnt cells (Figure 9A–C) when grown under normal (2.2 µM FA) conditions.

Following 3 weeks of culture in 0 µM FA, relative to 2.2 µM FA, both MDA-MB-231 and M-Wnt cells had ~40–50% reduced basal, maximal, and ATP synthase-coupled OCR (Figure 9D–F,H–J), and a ~1.7-fold increase in mitochondrial superoxide production (a marker of ETC dysfunction; Figure 9G,K).

## 4. Discussion

Treatment of TNBC remains challenging due to the lack of targeted therapies, as evidenced by the absence of formal guidelines for treatment of TNBC beyond cytotoxic chemotherapies [36]. Thus, new intervention targets and therapeutic strategies are urgently needed for TNBC. TNBCs exhibit several metabolic subtypes some of which are characterized by increased glycolysis [37], and mitochondrial dysfunction [38] which may inform therapeutic response and sensitivity to FA withdrawal. The antifolate methotrexate as a single agent in treatment of breast cancer has shown limited efficacy [39], due at least in part to two common issues with this class of drugs: toxicity and the development of resistance [13]. The former is addressed with supplementation of patients undergoing antifolate chemotherapy with high doses of leucovorin [13]. We have recently identified induction of type I interferon signaling as central to the transcriptomic response of M-Wnt cells to loss of FA [28], which may promote tumor growth via immune evasion [40]. However, the literature on the anticancer effects of dietary folate restriction is mixed. Multiple studies suggest an inhibitory effect of folate withdrawal on cancer cells in vitro and in vivo; conversely, folate supplementation shows a procancer effect under some conditions [29,41,42,43]. The literature is mixed, however, as several groups have reported withdrawal of FA promotes tumor invasiveness and EMT [44,45,46]. In the present study, we assessed the metabolic response and sensitivity to cytotoxic effects of FA restriction in TNBC, which has possible clinical implications for antifolate treatment. Specifically, we characterized the metabolic response to FA restriction in two TNBC models differing in metastatic potential and mitochondrial dysfunction.

MDA-MB-231 cells have a well-characterized mitochondrial respiratory chain defect that contributes to their metastatic phenotype [47,48], while M-Wnt cells have highly functional mitochondrial metabolism and low metastatic potential [31]. We found that while FA restriction enhanced mitochondrial dysfunction in both models of TNBC, FA restriction in the MDA-MB-231 model, relative to the M-Wnt TNBC model: (i) suppressed growth of orthotopically transplanted tumors; (ii) altered metabolomic profiles including one-carbon metabolism; and (iii) enhanced glycolysis and PPP metabolism. This suggests that the level of innate mitochondrial dysfunction may contribute to the responsiveness of TNBC cells to FA restriction, and extend the current literature indicating that disruption of one-carbon metabolism interacts with mitochondrial dysfunction to reprogram cancer-related metabolism [20,21,22,23,24,25,49,50].

The elevation of serine with concomitant reduction in several intermediates (such as AICAR; Figure 2D) of the de novo purine pathway in MDA-MB-231 tumors (but not M-Wnt tumors; Table S5) in response to FA restriction is indicative of impairment of one-carbon metabolism and nucleotide metabolism. This finding is not surprising since two steps of the de novo purine pathway require 10-formyltetrahydrofolate as the formyl donor. Response of two other one-carbon donors, serine and SAM, to dietary FA restriction is also readily traceable to folate metabolism. Serine conversion to glycine by SHMT1/2 provides substantial one-carbon groups to the folate pool [1]. Transfer of a methionine to ATP generates SAM, a universal methyl donor in the cell. While methionine, an essential amino acid, is mainly supplied by the diet, folate-dependent re-methylation of homocysteine is also important. Methionine, however, was increased in MDA-MB-231 (but not M-Wnt) tumors in response to FA-restricted diet. The observed drop in SAM may have arisen from insufficient ATP levels, coherent with inhibition of de novo purine pathway, and would explain the accumulation of methionine as the result of the decreased SAM biosynthesis. Likewise, decreased SAM is in agreement with the decrease in 5′-methyl thioadenosine as well as several products of the polyamine biosynthesis pathway.

Our observed change in metabolic gene expression in MDA-MB-231 (but not M-Wnt) tumors in response to FA restriction indicates MDA-MB-231 cells can compensate for the loss of folate-bound one-carbon transfer by enhancing the flux of the groups into the folate pool (elevation of MTHFD1 and SHMT1) or by re-directing them to specific metabolic pathways (elevation of MTHFR, ALDH1L1, and GNMT). There is substantial diversity among cancer cells in the utilization of cytosolic verses mitochondrial folate pathways, in response to reduced FA supply [51]. SHMT1/2 are two main sources of one-carbon groups for folate metabolism [1] and the two enzymes can compensate for each other [49]. For example, defects in mitochondrial folate metabolism in proliferating cells induce compensatory increases in cytoplasmic folate metabolism in part via SHMT1 [49]. Our data show that in response to such metabolic disruption, a compensatory increase in glucose metabolism takes place. Consistent with this finding, we previously showed in TNBC cell culture studies that folate withdrawal increased serine levels and reduced lactate levels [27]. Disruption of mitochondrial folate metabolism prompts metabolic adaptation such as enhanced cytosolic one-carbon metabolism, glycolysis, and PPP activity [21,49]. In the present study, alterations in levels of intermediates of glycolysis, accompanied by concomitant increased expression of several glycolytic enzymes and increased glucose uptake, indicate that MDA-MB-231 cells rely on glucose metabolism to meet their metabolic needs. Serine activates the critical glycolytic regulator PKM2 to promote glycolysis [52], and PKM2 plays a critical role in directing glycolysis to support rapid cell proliferation [53].

The mRNA expression of two genes encoding enzymes of folate metabolism often reduced in tumors, ALDH1L1 and GNMT, was markedly elevated in MDA-MB-231 (Figure 2L,O), but not M-Wnt tumors, from mice fed FA-restricted diet. These enzymes may be putative tumor suppressors, producing antiproliferative effects upon overexpression in cultured cancer cells [54,55]. ALDH1L1 is heterogeneously hypermethylated in many breast tumors [56], with high mRNA levels predicting favorable outcomes [57]. However, ALDH1L1 is hypomethylated in breast tumors following chemotherapy [58], and may be important for tumor cell survival in response to metabolic stress [59]. Thus, elevation of these enzymes could be yet another compensatory mechanism for tumors to survive nutrient starvation or other stressors by arresting proliferation or remodeling metabolism. Overall, we conclude that loss of dietary folate induces metabolic stress in MDA-MB-231 (but not M-Wnt) tumors, which is partially compensated for via glycolysis and PPP.

A common cause of increased glycolytic flux and PPP metabolism is respiratory deficiency [60]. Disruption of one-carbon metabolism can induce such mitochondrial dysfunction via suppression of mitochondrial protein translation, a phenomenon associated with two underlying mechanisms. First, initiation of mitochondrial translation requires folate-dependent formylation of methionyl-tRNA^met^ [21]. Mutations in methionyl-tRNA formyltransferase, the enzyme catalyzing this formylation, causes deficiency in oxidative phosphorylation [21]. Mammalian mitochondria also use folate-bound one-carbon groups, in the form of 5,10-methylene-THF, to produce taurinomethyluridine at the wobble position in mitochondrial tRNAs [20]. The lack of such methylation causes mitochondrial ribosome stalling at certain codons, which prevents translation and contributes to mitochondrial dysfunction [20]. Our in vitro models support these data; however, it is possible that cell line specific alterations in one-carbon metabolism might direct response to FA restriction independent of mechanisms identified here.

We also demonstrated that induction of glycolysis and the PPP in the MDA-MB-231 model is a compensatory mechanism in response to FA restriction and enhanced mitochondrial dysfunction. PPP metabolism is essential to cancer cell survival, particularly in response to elevated ROS production, a common occurrence in tumors. Of note, targeting PPP metabolism has been proposed as a cancer treatment strategy [61], since NADPH is critical in antioxidant defenses. Several cancer types, including TNBC, have been shown to depend heavily on NADPH derived from H6PD, an enzyme in the PPP, to provide the reducing equivalents required for DHFR activity, with loss of H6PD resulting in folate deficiency [62]. DHFR is responsible for the conversion of folic acid and dihydrofolate into the active coenzyme form THF. Thus, compensatory activation of the PPP in our experiments may in part be an attempt to refill the reduced folate pool by generating additional NADPH. The link between folate metabolism and NADPH, however, is bidirectional given that reactions of the folate pathway generate NADPH as well as consume in the cytoplasm [1]. In the mitochondria, however, folate metabolism can contribute significantly to the maintenance of cellular redox state by generating NADPH [22,25,63]. The importance of mitochondrial folate metabolism is further illustrated by the finding that cancer cells compensate for genetic disruption of mitochondrial folate metabolism via enhanced activity of cytosolic SHMT1 and reversal of the cytoplasmic metabolism to supply formate to the mitochondrial folate pool [49]. Similarly, increased de novo serine biosynthesis via PHGDH in breast cancer, where extracellular serine is limited, has a critical role for in vivo proliferation [64,65] and for the maintenance of mitochondrial redox homeostasis, and thereby cancer stemness [24].

Tumors derived from the M-Wnt model of TNBC demonstrated a different metabolomic and growth response to FA restriction than did the MDA-MB-231 tumor model. The latter model has been well characterized as bearing a dysfunctional ETC complex I, with decreased basal NAD(P)/NAD(P)H ratio [48], which may contribute to the sensitivity of MDA-MB-231 cells to disruption of folate metabolism by FA withdrawal. Disruption of cellular redox status via mitochondrial dysfunction has been shown to impair one-carbon metabolism and nucleotide production [66]. Indeed, in both methionine restriction-sensitive and -resistant TNBC cells, mitochondrial function is impaired by withdrawal of methionine, and successful adaptation requires remodeling of central-carbon metabolism and enhanced redox defense [50]. Accordingly, we hypothesized that intrinsic mitochondrial dysfunction may represent a decision point for cellular response to disruption of folate metabolism (i.e., extensively damaged mitochondria are beyond rescue by the PPP and/or the serine synthesis pathway, and thereby ruinous to the cell in the presence of glycolytic inhibitors). Our data indicate that intrinsic mitochondrial dysfunction in MDA-MB-231 cells results in a synthetic dependence on enhanced one-carbon, glycolytic, and PPP metabolism for survival. Indeed, metabolic rescue by glycolysis and/or the PPP upregulation appears to underpin an obligate adaptive response in MDA-MB-231 cells to disruptions of folate metabolism and increased ROS production. We postulate this is in part driven by the increased rate of reactive oxygen species production and mitochondrial dysfunction in these cells as compared with the M-Wnt cells, which are less sensitive to folate modulation. Metabolic plasticity in M-Wnt cells may enhance capacity to tolerate or even thrive in the presence of mitochondrial dysfunction in these cells, as evidenced by the enhanced tumor growth and invasiveness in FA-restricted conditions.

## 5. Conclusions

Our findings suggest that TNBC sensitivity to FA restriction may be informed by innate mitochondrial dysfunction, which can lead to decreased metabolic plasticity and increased dependence on one-carbon metabolism and glycolysis. Thus, folate deprivation or antifolate therapy following screening for TNBCs harboring high levels of mitochondrial dysfunction and associated metabolic inflexibility may represent a new precision-medicine approach. 

## Data Availability

The data presented in this study are available in Supplementary Tables S2, S4, and S5.

## Supplementary Materials

The following are available online at https://www.mdpi.com/article/10.3390/nu13051637/s1, Table S1: Primer sequences, Table S2: MDA-MB-231 tumor metabolomics, Table S3: MDA-MB-231 tumor metabolite VIP scores, Table S4: M-Wnt tumor metabolomics, Table S5: Liver metabolomics.

## References

[1] Ducker G.S., Rabinowitz J.D. (2017). One-Carbon Metabolism in Health and Disease. Cell Metab..

[2] Greenberg J.A., Bell S.J., Guan Y., Yu Y.-H. (2011). Folic Acid supplementation and pregnancy: More than just neural tube defect prevention. Rev. Obs. Gynecol..

[3] Keum N., Giovannucci E.L. (2014). Folic acid fortification and colorectal cancer risk. Am. J. Prev. Med..

[4] Lee J.E., Willett W.C., Fuchs C.S., Smith-Warner S.A., Wu K., Ma J., Giovannucci E. (2011). Folate intake and risk of colorectal cancer and adenoma: Modification by time. Am. J. Clin. Nutr..

[5] Rees J.R., Morris C.B., Peacock J.L., Ueland P.M., Barry E.L., McKeown-Eyssen G.E., Figueiredo J.C., Snover D.C., Baron J.A. (2017). Unmetabolized Folic Acid, Tetrahydrofolate, and Colorectal Adenoma Risk. Cancer Prev. Res..

[6] He L., Shen Y. (2017). MTHFR C677T polymorphism and breast, ovarian cancer risk: A meta-analysis of 19,260 patients and 26,364 controls. Oncol. Targets Ther..

[7] Maruti S.S., Ulrich C.M., Jupe E.R., White E. (2009). MTHFR C677T and postmenopausal breast cancer risk by intakes of one-carbon metabolism nutrients: A nested case-control study. Breast Cancer Res..

[8] Levine A.J., Figueiredo J.C., Lee W., Poynter J.N., Conti D., Duggan D.J., Campbell P.T., Newcomb P., Martinez M.E., Hopper J.L. (2010). Genetic variability in the MTHFR gene and colorectal cancer risk using the colorectal cancer family registry. Cancer Epidemiol. Biomark. Prev..

[9] Figueiredo J.C., Grau M.V., Haile R.W., Sandler R.S., Summers R.W., Bresalier R.S., Burke C.A., McKeown-Eyssen G.E., Baron J.A. (2009). Folic acid and risk of prostate cancer: Results from a randomized clinical trial. J. Natl. Cancer Inst..

[10] Ebbing M., Bønaa K.H., Nygård O., Arnesen E., Ueland P.M., Nordrehaug J.E., Rasmussen K., Njølstad I., Refsum H., Nilsen D.W. (2009). Cancer incidence and mortality after treatment with folic acid and vitamin B12. JAMA.

[11] Sauer J., Mason J.B., Choi S.W. (2009). Too much folate: A risk factor for cancer and cardiovascular disease?. Curr. Opin. Clin. Nutr. Metab. Care.

[12] Ulrich C.M., Potter J.D. (2007). Folate and cancer--timing is everything. JAMA.

[13] Visentin M., Zhao R., Goldman I.D. (2012). The antifolates. Hematol. Oncol. Clin. N. Am..

[14] Wahba H.A., El-Hadaad H.A. (2015). Current approaches in treatment of triple-negative breast cancer. Cancer Biol. Med..

[15] Wang S., Shi Y., Yuan Z., Wang X., Liu D., Peng R., Teng X., Qin T., Peng J., Lin G. (2012). Classical CMF regimen as adjuvant chemotherapy for triple-negative breast cancer may be more effective compared with anthracycline or taxane-based regimens. Med. Oncol..

[16] Nasr K.E., Osman M.A., Elkady M.S., Ellithy M.A. (2015). Metronomic methotrexate and cyclophosphamide after carboplatin included adjuvant chemotherapy in triple negative breast cancer: A phase III study. Ann. Transl. Med..

[17] Bianchini G., Balko J.M., Mayer I.A., Sanders M.E., Gianni L. (2016). Triple-negative breast cancer: Challenges and opportunities of a heterogeneous disease. Nat. Rev. Clin. Oncol..

[18] Dent R., Hanna W.M., Trudeau M., Rawlinson E., Sun P., Narod S.A. (2009). Pattern of metastatic spread in triple-negative breast cancer. Breast Cancer Res. Treat..

[19] Liedtke C., Mazouni C., Hess K.R., Andre F., Tordai A., Mejia J.A., Symmans W.F., Gonzalez-Angulo A.M., Hennessy B., Green M. (2008). Response to neoadjuvant therapy and long-term survival in patients with triple-negative breast cancer. J. Clin. Oncol. Off. J. Am. Soc. Clin. Oncol..

[20] Morscher R.J., Ducker G.S., Li S.H., Mayer J.A., Gitai Z., Sperl W., Rabinowitz J.D. (2018). Mitochondrial translation requires folate-dependent tRNA methylation. Nature.

[21] Minton D.R., Nam M., McLaughlin D.J., Shin J., Bayraktar E.C., Alvarez S.W., Sviderskiy V.O., Papagiannakopoulos T., Sabatini D.M., Birsoy K. (2018). Serine Catabolism by SHMT2 Is Required for Proper Mitochondrial Translation Initiation and Maintenance of Formylmethionyl-tRNAs. Mol. Cell.

[22] Piskounova E., Agathocleous M., Murphy M.M., Hu Z., Huddlestun S.E., Zhao Z., Leitch A.M., Johnson T.M., DeBerardinis R.J., Morrison S.J. (2015). Oxidative stress inhibits distant metastasis by human melanoma cells. Nature.

[23] Kim D., Fiske B.P., Birsoy K., Freinkman E., Kami K., Possemato R.L., Chudnovsky Y., Pacold M.E., Chen W.W., Cantor J.R. (2015). SHMT2 drives glioma cell survival in ischaemia but imposes a dependence on glycine clearance. Nature.

[24] Samanta D., Park Y., Andrabi S.A., Shelton L.M., Gilkes D.M., Semenza G.L. (2016). PHGDH Expression Is Required for Mitochondrial Redox Homeostasis, Breast Cancer Stem Cell Maintenance, and Lung Metastasis. Cancer Res..

[25] Yang L., Garcia Canaveras J.C., Chen Z., Wang L., Liang L., Jang C., Mayr J.A., Zhang Z., Ghergurovich J.M., Zhan L. (2020). Serine Catabolism Feeds NADH when Respiration Is Impaired. Cell Metab..

[26] Fox J.T., Stover P.J. (2008). Folate-mediated one-carbon metabolism. Vitam. Horm..

[27] Ashkavand Z., O’Flanagan C., Hennig M., Du X., Hursting S.D., Krupenko S.A. (2017). Metabolic Reprogramming by Folate Restriction Leads to a Less Aggressive Cancer Phenotype. Mol. Cancer Res..

[28] Kok D.E., O’Flanagan C.H., Coleman M.F., Ashkavand Z., Hursting S.D., Krupenko S.A. (2020). Effects of folic acid withdrawal on transcriptomic profiles in murine triple-negative breast cancer cell lines. Biochimie.

[29] Oleinik N.V., Helke K.L., Kistner-Griffin E., Krupenko N.I., Krupenko S.A. (2014). Rho GTPases RhoA and Rac1 mediate effects of dietary folate on metastatic potential of A549 cancer cells through the control of cofilin phosphorylation. J. Biol. Chem..

[30] Hansen M.F., Jensen S.O., Fuchtbauer E.M., Martensen P.M. (2017). High folic acid diet enhances tumour growth in PyMT-induced breast cancer. Br. J. Cancer.

[31] O’Flanagan C.H., Rossi E.L., McDonell S.B., Chen X., Tsai Y.H., Parker J.S., Usary J., Perou C.M., Hursting S.D. (2017). Metabolic reprogramming underlies metastatic potential in an obesity-responsive murine model of metastatic triple negative breast cancer. NPJ Breast Cancer.

[32] Wang X., Spandidos A., Wang H., Seed B. (2011). PrimerBank: A PCR primer database for quantitative gene expression analysis, 2012 update. Nucleic Acids Res..

[33] Livak K.J., Schmittgen T.D. (2001). Analysis of relative gene expression data using real-time quantitative PCR and the 2(-Delta Delta C(T)) Method. Methods.

[34] Dunlap S.M., Chiao L.J., Nogueira L., Usary J., Perou C.M., Varticovski L., Hursting S.D. (2012). Dietary energy balance modulates epithelial-to-mesenchymal transition and tumor progression in murine claudin-low and basal-like mammary tumor models. Cancer Prev. Res..

[35] Shane B. (2011). Folate status assessment history: Implications for measurement of biomarkers in NHANES. Am. J. Clin. Nutr..

[36] Fornier M., Fumoleau P. (2012). The paradox of triple negative breast cancer: Novel approaches to treatment. Breast J..

[37] Gong Y., Ji P., Yang Y.S., Xie S., Yu T.J., Xiao Y., Jin M.L., Ma D., Guo L.W., Pei Y.C. (2021). Metabolic-Pathway-Based Subtyping of Triple-Negative Breast Cancer Reveals Potential Therapeutic Targets. Cell Metab..

[38] Pelicano H., Zhang W., Liu J., Hammoudi N., Dai J., Xu R.H., Pusztai L., Huang P. (2014). Mitochondrial dysfunction in some triple-negative breast cancer cell lines: Role of mTOR pathway and therapeutic potential. Breast Cancer Res..

[39] Fracchia A.A., Farrow J.H., Adam Y.G., Monroy J., Knapper W.H. (1970). Systemic chemotherapy for advanced breast cancer. Cancer.

[40] Chen J., Cao Y., Markelc B., Kaeppler J., Vermeer J.A.F., Muschel R.J. (2019). Type I IFN protects cancer cells from CD8^+^ T cell–mediated cytotoxicity after radiation. J. Clin. Investig..

[41] Tomaszewski J.J., Cummings J.L., Parwani A.V., Dhir R., Mason J.B., Nelson J.B., Bacich D.J., O’Keefe D.S. (2011). Increased cancer cell proliferation in prostate cancer patients with high levels of serum folate. Prostate.

[42] Rycyna K.J., Bacich D.J., O’Keefe D.S. (2013). Opposing roles of folate in prostate cancer. Urology.

[43] Strickland K.C., Krupenko N.I., Krupenko S.A. (2013). Molecular mechanisms underlying the potentially adverse effects of folate. Clin. Chem. Lab. Med..

[44] Su Y.H., Huang W.C., Huang T.H., Huang Y.J., Sue Y.K., Huynh T.T., Hsiao M., Liu T.Z., Wu A.T., Lin C.M. (2016). Folate deficient tumor microenvironment promotes epithelial-to-mesenchymal transition and cancer stem-like phenotypes. Oncotarget.

[45] Wang T.P., Hsu S.H., Feng H.C., Huang R.F. (2012). Folate deprivation enhances invasiveness of human colon cancer cells mediated by activation of sonic hedgehog signaling through promoter hypomethylation and cross action with transcription nuclear factor-kappa B pathway. Carcinogenesis.

[46] Liu Z., Jin X., Pi W., Liu S. (2017). Folic acid inhibits nasopharyngeal cancer cell proliferation and invasion via activation of FRalpha/ERK1/2/TSLC1 pathway. Biosci. Rep..

[47] Ishikawa K., Takenaga K., Akimoto M., Koshikawa N., Yamaguchi A., Imanishi H., Nakada K., Honma Y., Hayashi J. (2008). ROS-generating mitochondrial DNA mutations can regulate tumor cell metastasis. Science.

[48] Santidrian A.F., Matsuno-Yagi A., Ritland M., Seo B.B., LeBoeuf S.E., Gay L.J., Yagi T., Felding-Habermann B. (2013). Mitochondrial complex I activity and NAD+/NADH balance regulate breast cancer progression. J. Clin. Investig..

[49] Ducker G.S., Chen L., Morscher R.J., Ghergurovich J.M., Esposito M., Teng X., Kang Y., Rabinowitz J.D. (2016). Reversal of Cytosolic One-Carbon Flux Compensates for Loss of the Mitochondrial Folate Pathway. Cell Metab..

[50] Borrego S.L., Fahrmann J., Datta R., Stringari C., Grapov D., Zeller M., Chen Y., Wang P., Baldi P., Gratton E. (2016). Metabolic changes associated with methionine stress sensitivity in MDA-MB-468 breast cancer cells. Cancer Metab..

[51] Lee W.D., Pirona A.C., Sarvin B., Stern A., Nevo-Dinur K., Besser E., Sarvin N., Lagziel S., Mukha D., Raz S. (2021). Tumor Reliance on Cytosolic versus Mitochondrial One-Carbon Flux Depends on Folate Availability. Cell Metab..

[52] Chaneton B., Hillmann P., Zheng L., Martin A.C.L., Maddocks O.D.K., Chokkathukalam A., Coyle J.E., Jankevics A., Holding F.P., Vousden K.H. (2012). Serine is a natural ligand and allosteric activator of pyruvate kinase M2. Nature.

[53] Dayton T.L., Jacks T., Vander Heiden M.G. (2016). PKM2, cancer metabolism, and the road ahead. EMBO Rep..

[54] DebRoy S., Kramarenko I.I., Ghose S., Oleinik N.V., Krupenko S.A., Krupenko N.I. (2013). A novel tumor suppressor function of glycine N-methyltransferase is independent of its catalytic activity but requires nuclear localization. PLoS ONE.

[55] Krupenko S.A., Krupenko N.I. (2018). ALDH1L1 and ALDH1L2 Folate Regulatory Enzymes in Cancer. Adv. Exp. Med. Biol..

[56] Beniaminov A.D., Puzanov G.A., Krasnov G.S., Kaluzhny D.N., Kazubskaya T.P., Braga E.A., Kudryavtseva A.V., Melnikova N.V., Dmitriev A.A. (2018). Deep Sequencing Revealed a CpG Methylation Pattern Associated With ALDH1L1 Suppression in Breast Cancer. Front. Genet..

[57] Wu S., Xue W., Huang X., Yu X., Luo M., Huang Y., Liu Y., Bi Z., Qiu X., Bai S. (2015). Distinct prognostic values of ALDH1 isoenzymes in breast cancer. Tumour Biol..

[58] Luo Y., Huang J., Tang Y., Luo X., Ge L., Sheng X., Sun X., Chen Y., Zhu D. (2019). Regional methylome profiling reveals dynamic epigenetic heterogeneity and convergent hypomethylation of stem cell quiescence-associated genes in breast cancer following neoadjuvant chemotherapy. Cell Biosci..

[59] Lee S.-H., Jeon Y., Kang J.H., Jang H., Lee H., Kim S.-Y. (2020). The Combination of Loss of ALDH1L1 Function and Phenformin Treatment Decreases Tumor Growth in KRAS-Driven Lung Cancer. Cancers.

[60] Gohil V.M., Sheth S.A., Nilsson R., Wojtovich A.P., Lee J.H., Perocchi F., Chen W., Clish C.B., Ayata C., Brookes P.S. (2010). Nutrient-sensitized screening for drugs that shift energy metabolism from mitochondrial respiration to glycolysis. Nat. Biotechnol..

[61] Cho E.S., Cha Y.H., Kim H.S., Kim N.H., Yook J.I. (2018). The Pentose Phosphate Pathway as a Potential Target for Cancer Therapy. Biomol. Ther..

[62] Chen L., Zhang Z., Hoshino A., Zheng H.D., Morley M., Arany Z., Rabinowitz J.D. (2019). NADPH production by the oxidative pentose-phosphate pathway supports folate metabolism. Nat. Metab..

[63] Fan J., Ye J., Kamphorst J.J., Shlomi T., Thompson C.B., Rabinowitz J.D. (2014). Quantitative flux analysis reveals folate-dependent NADPH production. Nature.

[64] Sullivan M.R., Mattaini K.R., Dennstedt E.A., Nguyen A.A., Sivanand S., Reilly M.F., Meeth K., Muir A., Darnell A.M., Bosenberg M.W. (2019). Increased Serine Synthesis Provides an Advantage for Tumors Arising in Tissues Where Serine Levels Are Limiting. Cell Metab..

[65] Locasale J.W., Grassian A.R., Melman T., Lyssiotis C.A., Mattaini K.R., Bass A.J., Heffron G., Metallo C.M., Muranen T., Sharfi H. (2011). Phosphoglycerate dehydrogenase diverts glycolytic flux and contributes to oncogenesis. Nat. Genet..

[66] Diehl F.F., Lewis C.A., Fiske B.P., Vander Heiden M.G. (2019). Cellular redox state constrains serine synthesis and nucleotide production to impact cell proliferation. Nat. Metab..

